# Expression of Concern: Evaluating the Clinical Efficacy of an Exergame-Based Training Program for Enhancing Physical and Cognitive Functions in Older Adults With Mild Cognitive Impairment and Dementia Residing in Rural Long-Term Care Facilities: Randomized Controlled Trial

**DOI:** 10.2196/75355

**Published:** 2025-04-04

The publisher expresses concern regarding the following article:

Evaluating the Clinical Efficacy of an Exergame-Based Training Program for Enhancing Physical and Cognitive Functions in Older Adults With Mild Cognitive Impairment and Dementia Residing in Rural Long-Term Care Facilities: Randomized Controlled Trial [1].

This article is under investigation for potential peer review irregularities. Readers are advised to interpret the findings with caution pending the outcome of this inquiry.
